# Investigation of SARS-CoV-2 individual proteins reveals the in vitro and in vivo immunogenicity of membrane protein

**DOI:** 10.1038/s41598-023-49077-2

**Published:** 2023-12-18

**Authors:** Timothy Haystead, Eric Lee, Kirstin Cho, Gail Gullickson, Philip Hughes, Greta Krafsur, Robert Freeze, Scott Scarneo

**Affiliations:** 1 EydisBio Inc, Durham, NC 27701 USA; 2 Department of Pharmacology and Cancer Biology, Duke University School of Medicine, Durham, NC 27701 USA; 3 Inotiv Inc, Boulder, CO USA

**Keywords:** Cytokines, Infection, Inflammation

## Abstract

Evidence in SARS-CoV-2 patients have identified that viral infection is accompanied by the expression of inflammatory mediators by both immune and stromal cells within the pulmonary system. However, the immunogenicity of individual SARS-CoV-2 proteins has yet to be evaluated. The SARS-CoV-2 virus consists of 29 proteins, categorized either as nonstructural proteins (NSP’s), structural proteins (SP’s) or accessory proteins. Here we sought to evaluate the immunogenicity of NSP 1, 7, 8, 9, 10, 12, 14, 16 and the SP’s spike protein (full length, S1, S2 and receptor binding domain subunits), nucleocapsid and membrane SARS-CoV-2 proteins against THP-1 and human peripheral blood mononuclear cells (PBMCs). Our results indicate that various SARS-CoV-2 proteins elicit a proinflammatory immune response indicated by increases in cytokines TNF, IL-6 and IL-1β. Our results support that SARS-CoV-2 membrane protein induced a robust increase of TNF, IL-6, IL-1β and IL-10 expression in both THP-1 and human PBMC’s. Further evaluation of intranasal membrane protein challenge in male and female BALB/c mice show increases in BALF (bronchalveolar lavage fluid) proinflammatory cytokine expression, elevated cellularity, predominantly neutrophilic, and concomitant peribronchiolar and perivascular lymphomononuclear and neutrophilic inflammation. Our results suggest that individual membrane associated SARS-CoV-2 proteins induce a robust immune response that may contribute to viral induced cytokine release syndrome (CRS) in the lungs of moderate to severe COVID-19 patients. We posit that SARS-CoV-2 membrane challenges in immune-competent mice can serve as an adequate surrogate for the development of novel treatments for SARS-CoV-2 induced pulmonary inflammation, thereby avoiding expensive live virus studies under BSL-3 conditions.

## Introduction

Since the start of the 2020 SARS-CoV-2 pandemic, great advances in our basic understanding of SARS-CoV-2 virology and subsequent development of anti-viral therapeutics have occurred. Despite these advancements, there still remains a large unmet need to develop novel therapeutics that adequately address the underlying pathology observed in moderate to severe COVID-19 patients^1–3^. Clinical data in these patients has shown that viral-induced pulmonary inflammation is a major complication and indicator of poor outcomes^4,5^. The release of cytokines within the pulmonary system in response to viral infection can lead to cytokine release syndrome (CRS) in which patients exhibit a maladaptive increase in inflammatory cytokines such as TNF, IL-6 and IL-1β within the lungs^6,7^. Increases in proinflammatory cytokines lead to the infiltration of neutrophils, macrophages, T cells, B cells and other immune cells which in turn can create a feed forward mechanism exacerbating inflammation. Additionally, the infiltration of immune cells into the pulmonary bronchoalveolar space can significantly impair gas exchange leading to decreased oxygen levels in patients. Although the release of inflammatory cytokines is a critical process for the innate and adaptive immune response to viral infection, over activation, (i.e., greater than that needed to prime the proper immune response) leads to excessive tissue damage, associated with infiltration of immune cells and persistent inflammation even past viral clearance.

Since the onset of the COVID-19 pandemic, there have been limited in vivo models of SARS-CoV-2. The most used K18 humanized ACE2 mouse model, allows for viral replication, but induces rapid death and the viral inflammatory process has been poorly described. Furthermore, this mouse model requires a Bio Safety Level 3 (BSL-3) facility which greatly limits its use due to costs and availability of these facilities. Other models, such as the Syrian hamster model, do not induce rapid death and fail to produce robust cytokine production. Furthermore, the Syrian hamster model is limited by lack of validated reagents targeted towards hamster tissues/cells to study immune system responses. Thus, novel non-BSL-3 facility animal models that adequately induce pulmonary inflammation are needed to expand our repertoire of pre-clinical models.

Expression of individual SARS-CoV-2 proteins and their immunogenicity may contribute to COVID-19 pulmonary hyperinflammation. During viral replication, it has been shown that individual viral proteins may be released either when an infected cell lyses or following tissue damage. The release of both packaged and unpackaged viral proteins into the pulmonary space can stimulate the immune system leading to inflammation within the pulmonary tissues. These proteins can elicit pathogen-associated molecular patterns (PAMPs) within the pulmonary tissues leading to increased immune infiltration and cytokine expression^8^. Although, to date, the specific molecular response to these proteins has yet to be studied.

Here, we evaluate the inflammatory response of 14 SARS-CoV-2 proteins consisting of 8 non-structural proteins (NSP’s) including NSP 1, 7, 8, 9, 10, 12, 14, and 16 as well as structural proteins (SP’s) including membrane, nucleocapsid and full spike protein, the S1, S2 and receptor binding domain (RBD) of the spike protein, in differentiated human THP-1 cells. Further evaluation of membrane protein in male and female human peripheral mononuclear cells (PBMC’s) showed a significant increase in TNF, IL-6, IL-1β and IL-10 expression. In vivo intranasal challenge with SARS-CoV-2 membrane protein showed significant increase in infiltrating immune cells, inflammatory cytokine expression in bronchiolar alveolar lavage fluid (BALF) as well as histological changes consistent with pulmonary inflammation.

## Materials and methods

### THP-1 cell culture

THP-1 cells (ATCC-TIB-202) were thawed and plated in RPMI media containing 10% FBS, 50 μM 2-mercaptoethanol, 1% Penn/Strep/Glutamax. Following 1–5 passages, cells were collected, pelleted, washed and counted. Cells were seeded into a 24 well tissue culture treated plate at 10,000 cells/well in 0.5 ml of media. THP-1cells were treated with 100 nM phorbol 12-myristate 13-acetate (PMA) for 72 h in media. After 72 h PMA was washed off and cells will be cultured for 48 h in 0.5 ml media without PMA. On Day 5, cells were stimulated with 10 ng/ml LPS positive control (Sigma-L4391-1MG), SARS-CoV-2 proteins or unstimulated. Cells were cultured for 24 h, and supernatant collected for cytokine analysis. Vendors and catalogue numbers for all SARS-CoV-2 proteins used are noted in Table 1.

**Table 1:** Reagents.

Protein	Vendor	Catalogue number
SARS-CoV-2 Nsp1	R&D Systems	10666-CV-050
SARS-CoV-2 Nsp7	R&D Systems	10632-CV-100
SARS-CoV-2 Nsp8	R&D Systems	10633-CV-100
SARS-CoV-2 Nsp9	R&D Systems	10631-CV-100
SARS-CoV-2 Nsp10	R&D Systems	10630-CV-100
SARS-CoV-2 Nsp12	R&D Systems	10686-CV-050
SARS-CoV-2 Nsp14	R&D Systems	10667-CV-050
SARS-CoV-2 Nsp16	R&D Systems	10634-CV-100
SARS-CoV-2 Membrane	Novus	NBP3-07080
SARS-CoV-2 Membrane	Innovative Research	ICOV2MBRHIS50UG
SARS-CoV-2 Spike Protein S1 Subunit	R&D Systems	10569-CV-100
SARS-CoV-2 Spike Protein S2 Subunit	R&D Systems	10594-CV-100
SARS-CoV-2 Spike Protein RBD Subunit	R&D Systems	10500-CV-100
SARS-CoV-2 Spike Protein Full Length	R&D Systems	10549-CV-100
SARS-CoV-2 Nucleocapsid	R&D Systems	10474-CV-050

### Human PBMC’s

Human PBMC’s isolated from healthy volunteers, ZenBio (SER-PBMC-F), cells were thawed in RPMI media containing 10% FBS and 1% Penn/Strep/Glutamax. Cells were stimulated for 24 h in the presence of varying concentrations of membrane protein, vehicle (DPBS, Corning-21-031-CV) and LPS positive control (Sigma-L4391-1MG). Cell culture supernatant was collected and stored at − 80 F for future cytokine analysis.

### Cytokine analysis

Serum, BALF and cell culture samples were analyzed by Luminex cytokine panels for TNF, IL-1β, IL-4, IL-6, IL-10, CCL2, IFNγ. 200 μl of wash buffer was added into each well of the plate. The wash buffer was sealed and mixed on a plate shaker for 10 min at room temperature (20–25 °C). Wash buffer was decanted and the residual amount removed from all wells by inverting the plate and tapping it smartly onto absorbent towels several times. 50 μl of each standard or control was added into the appropriate wells. The serum matrix was used for 0 pg/ml standard (Background). 25 μl of assay buffer was added to the sample wells. 25 μl of sample was added into the appropriate wells. Mixing bottle was vortexed and 25 μl of the mixed or premixed beads added to each well. The plate was sealed with a plate sealer. The plate was wrapped with foil and incubated with agitation on a plate shaker overnight (16–18 h) at 2–8 °C. Well contents were gently removed and plate washed 3 times following kit instructions. 25 μl of detection antibodies were added into each well. The well was sealed, covered with foil and incubated with agitation on a plate shaker for 1 h at room temperature (20–25 °C). 25 μl of Streptavidin–Phycoerythrin was added to each well containing the 25 μl of detection antibodies. The wells were sealed, covered with foil and incubated with agitation on a plate shaker for 30 min at room temperature (20–25 °C). Well contents were gently removed and the plate washed 3 times following kit instructions. 150 μl of Sheath Fluid was added to all wells. The beads were resuspended on a plate shaker for 5 min, and the plate run on Luminex 200TM. Absolute expression was determined by standard curve of known analyte expression. Data expressed as cytokine concentration (pg/ml).

### Animal care

Male and female BALB/c mice were purchased from Jackson Labratories (Bar Harbor, Maine) at 9 weeks old. Mice were accommodated in a facility where temperature and humidity were controlled, and they followed a 12-h light/dark cycle (with lights turning on at 7 a.m.), with unrestricted access to food and water. All experiments were carried out in accordance of Inotiv Boulder Institution Animal Care and Use Committee (IACUC) and approved by Inotiv’s institutional animal care and use committee under IACUC protocol No. IB-071. All studies conformed to the National Institutes of Health Guide for the Care and Use of Laboratory Animals. All experiments followed current ARRIVE (Animal Research: Reporting of In Vivo Experiments) guidelines.

### BALB/c intranasal membrane challenge

On study day-1, animals were randomized into treatment groups based on body weight. On day 0, animals receiving intranasal administration were briefly anesthetized with 3% isoflurane and 50 μl of membrane protein or PBS (DPBS, Corning-21-031-CV) was instilled into the nose dropwise via a pipette. Animals were kept in an upright position for 2 min to allowed for the fluid to trickle into the lungs; afterwards, animals were returned to their home cage for recovery. Clinical observations were made of animals in the 24 h following intranasal challenge. 24 h post-intranasal challenge, all animals were euthanized by CO_2_ affixation. After euthanasia, a 20 g blunt tipped needle was inserted into the trachea, the lungs washed with 0.8 ml of sterile saline 3 times, the samples were pooled into a 15 ml flip-top conical and kept on ice.

### Histopathology

Histopathologic evaluation was performed on whole lung specimens from all animals. After the lung lavage, 0.8 ml of neutral buffered formalin (NBF) was infused into the lung for inflation before placing into the NBF jar for histopathology. Lung samples were processed and embedded whole, sectioned longitudinally (to include major airways) for maximum area visualization, and stained with hematoxylin and eosin (H&E) for pathology evaluation. The entire lung section was evaluated for the following parameters. Lungs were scored for peribronchiolar lymphoid inflammation as described in Histopathology scoring. The widths of lymphoid cell infiltrates of five of the most severely-affected areas were measured, and a mean was determined.

### Histopathology scoring

#### Peribronchiolar Lymphoid Inflammation Scoring Criteria Score

0 = Normal, 0.5 ≤ 25 microns, 1 = 26–50 microns, 2 = 51–75 microns, 3 = 76–100 microns, 4 = 101–150 microns, 5 ≥ 150 microns.

#### Neutrophil Percent and Neutrophil Score

Inflammatory cell infiltrates were evaluated for approximate percent of neutrophils in the total infiltrate, rounded to 0, 10, 25, 50, or 75%. This value was then multiplied by the inflammation score to determine the neutrophil score.

#### Interstitial Inflammation Score

The approximate percentage of lung affected by inflammation was evaluated and scored as follows. 0 = None, 0.5 ≤ 5%, 1 = 6–10%, 2 = 11–15%, 3 = 26–50%, 4 = 51–75%, 5 ≥ 75%.

#### Airway Epithelial Damage Score

Lungs were scored for the degree of airway epithelial damage, according to the worst lesion present, as follows. 0 = None; normal intact airway epithelium, 1 = Elongation and distortion of the cuboidal/columnar epithelial cells lining the airways, 2 = Elongation associated with in folding of the epithelium and narrowing of the airway lumen, 3 = Loss of epithelial cells resulting in broken airways.

### Statistical analysis

Graphpad Prism 9 was used for all statistical tests throughout the study. All statistical tests for data presented are mentioned in the figure legends.

## Results

**Figure 1:**
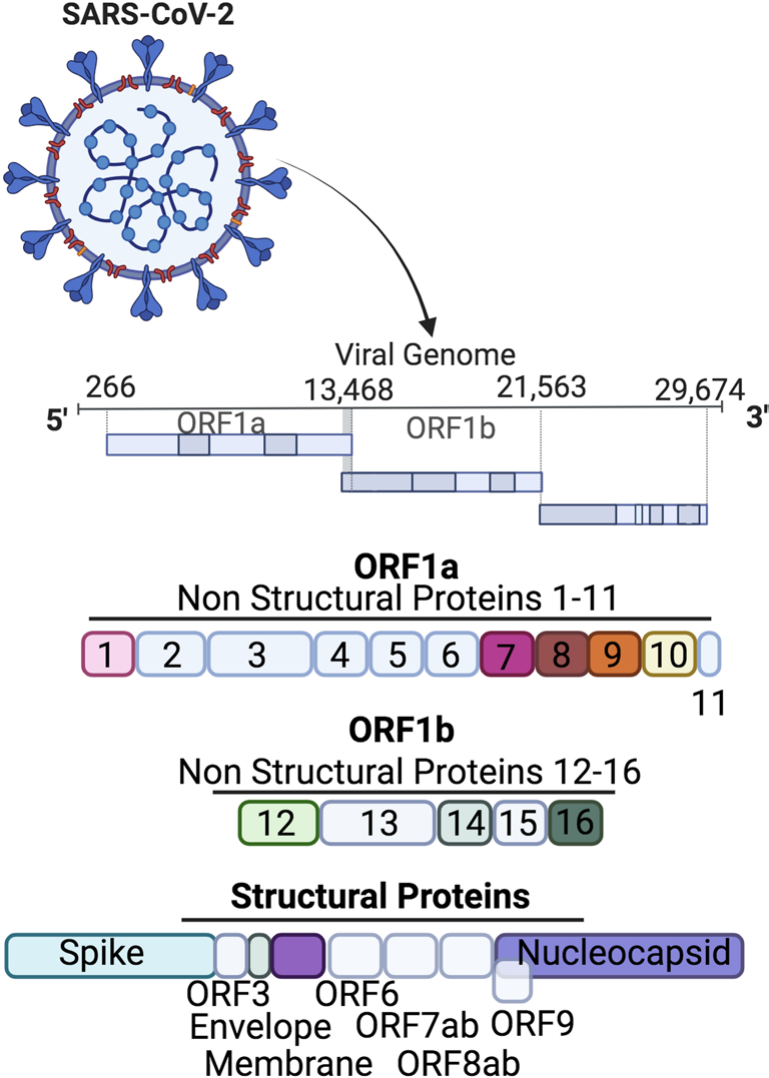
Overview of the 29 distinct SARS-CoV-2 proteins including NSP’s and SP’s. The SARS-CoV-2 virus consists of 29 distinct proteins comprising nonstructural proteins involved in viral replication and viral processes as well as structural proteins which include proteins directly responsible to virion structure.

**Figure 2:**
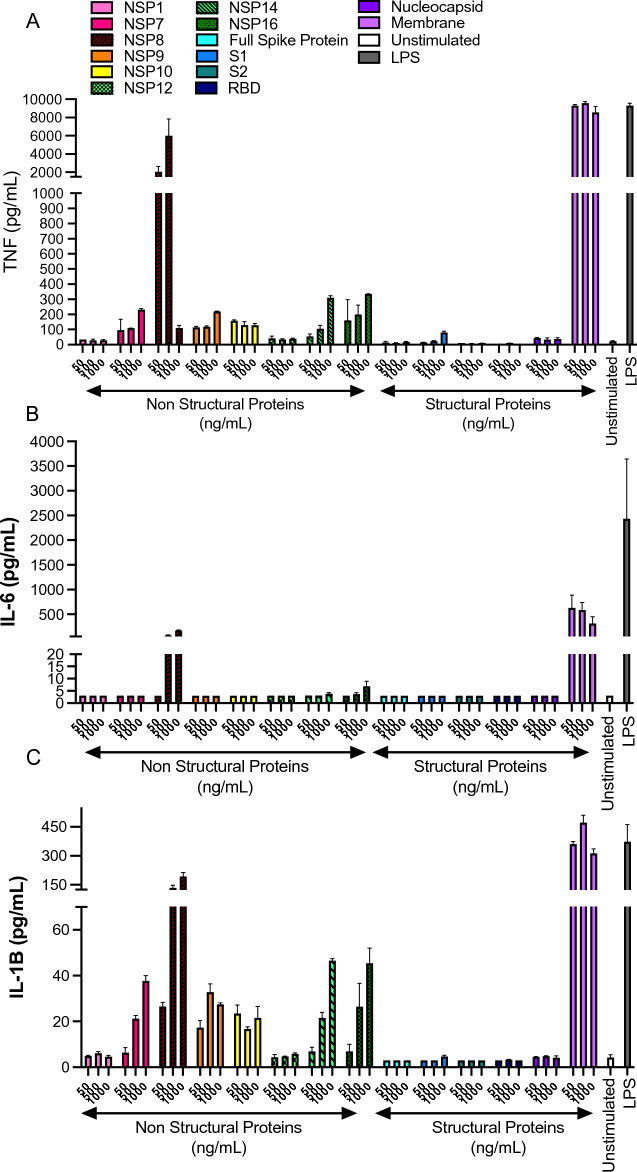
SARS-CoV-2 proteins induce TNF, IL-6 and IL-1β expression. Following THP-1 macrophage differentiation with PMA (100 nM) for 72 h, cells were rested for another 38 h in PMA-free media. Adherent THP-1 cells were treated with [50–1000 ng/ml] of purified NSPs or SPs (spike, nucleocapsid and membrane) for 24 h. (**A**) Expression of TNF (pg/ml), (**B**) IL-6 (pg/ml) and (**C**) IL-1β (pg/ml) expression levels in supernatant were determined as well as TNF, IL-6 and IL-1β expression in unstimulated cells (negative control) and LPS 10 ng/ml (positive control). N = 2/condition Data presented as mean ± SD per titration dose.

We conducted an in vitro screen of SARS-CoV-2 NSP’s and SP’s on cytokine expression in fully differentiated THP-1 cells. Of the 29 distinct SARS-CoV-2 proteins encoded for in the viral genome, we tested 14 including the S1,S2 and RBD of the spike protein (Fig. 1). Following THP-1 macrophage differentiation with PMA, adherent THP-1 cells were treated with [50–1000 ng/ml] of purified NSP’s or SP’s (spike, nucleocapsid and membrane) for 24 h. Following stimulation, supernatants were collected, and cytokine expression quantified by Luminex (Fig. 2A–C). Out of the 14 proteins studied, NSP 7, 8, 9, 14, 16 and membrane proteins showed significant upregulation of TNF secretion. Interestingly membrane protein showed saturated TNF response at all treatment concentrations (~ 8000 pg/ml TNF) (Fig. 2A). IL-6 concentrations were only upregulated by 3 proteins including NSP 8, 16 and membrane protein (Fig. 2B). Interestingly, IL-1β concentrations were upregulated in 7 SARS-CoV-2 proteins, including NSP 7, 8, 9, 10, 14, 16 and membrane (Fig. 2C). Due to the saturating response of SARS-CoV-2 membrane protein on TNF, IL-6 and IL-1β cytokine expression, we next sought to validate this response with separate vendor-sourced membrane protein (Novus Biologicals and Tocris) to confirm the response was not the result of any potential endotoxin contamination from a specific batch/lot of purified protein. Both Novus Biological and Innovative Research sourced purified membrane protein showed a robust and similar response on TNF secretion (Supplemental Fig. 1A). Furthermore, membrane protein stimulation at [5000–50 ng/ml] in human PBMC’s did not significantly affect cell viability (Supplemental Fig. 1B).

**Figure 3:**
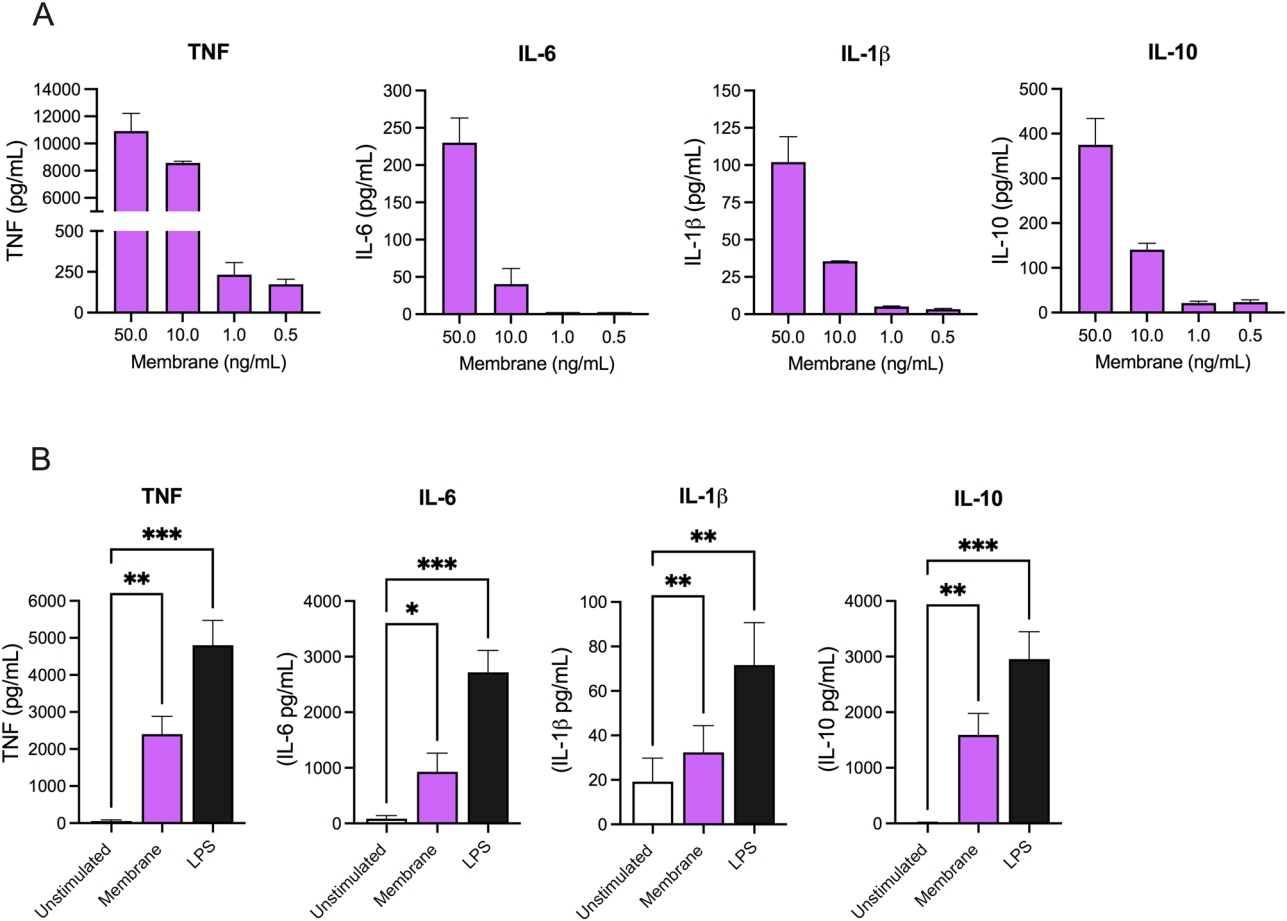
SARS-CoV-2 membrane effects on THP-1 cells and healthy human PBMC’s. (**A**) THP-1 cytokine expression following dose response titration of membrane protein [0.5–50.0 ng/ml] in THP-1 cells. N = 2/condition Data presented as mean ± SD per titration dose. (**B**) Human PBMC’s were stimulated with membrane protein at 50 ng/ml or LPS (10 ng/ml, positive control) for 24 h. Expression of TNF, IL-6, IL-1β and IL-10 were reported. N = 6 3M + 3F per group. Data represents mean ± SEM. *p < 0.05, **p < 0.01, **p < 0.001. Data analyzed by 1-way ANOVA with Dunnett’s multiple comparison test.

Following the observation that treatment of THP-1 cells with SARS-CoV-2 membrane protein at 50–1000 ng/ml showed no dose-dependent changes most likely due to saturation of cellular responses, we next titrated down the concentration of membrane protein to observe the potency of membrane protein on downstream cytokine response. [0.5–50 ng/ml] of membrane protein was used to stimulate differentiated THP-1 cells (Fig. 3A). Membrane treated THP-1 cells showed TNF response down to 500 pg/ml of membrane protein whereas IL-6 required 10 ng/ml concentration to stimulate a significant response. No response was observed in IFNγ and IL-4 expression levels following membrane protein challenge (Supplemental Fig. 2).

To further validate the immunological response to SARS-CoV-2 membrane protein, we next evaluated the cytokine response in healthy human PBMC’s. Here, PBMC’s from 6 healthy volunteers (3M + 3F) were treated with membrane protein at 50 ng/ml concentration for 24 h. TNF, IL-6, IL-1β and IL-10 cytokine expression was significantly upregulated in PBMCs compared to vehicle-treated cells (Fig. 3B).

**Figure 4:**
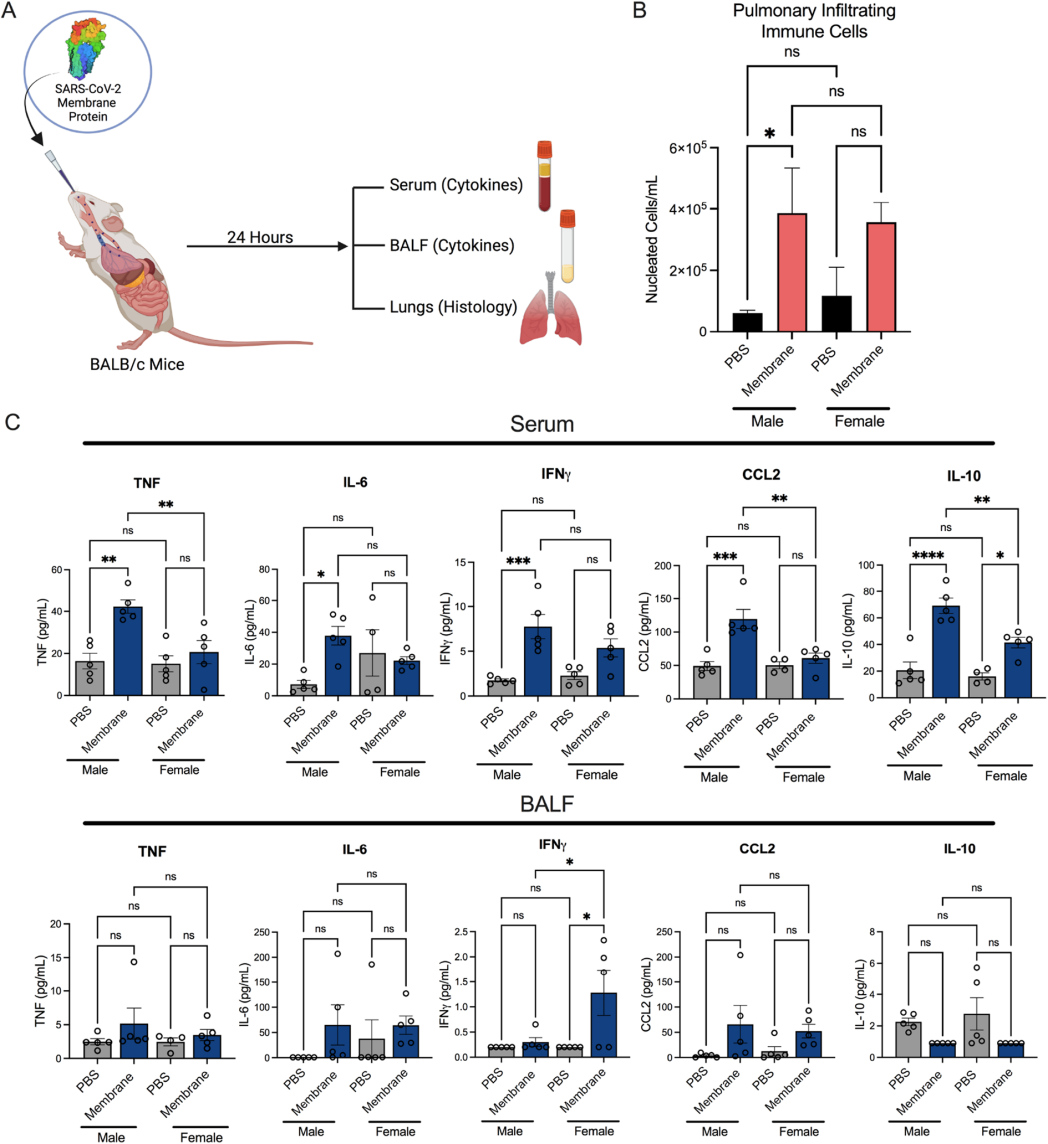
Serum and BALF cytokine expression between PBS and membrane intranasally challenged male and female BALB/c mice. (**A**) Male and female BALB/c mice were challenged intranasally with 15 μg of purified SARS-CoV-2 membrane protein for 24 h. Serum and BALF samples were collected for cytokine analysis/ immune cell evaluation. Pulmonary tissues were collected for pathohistological analysis. (**B**) Number of immune cells per mL of BALF samples in vehicle and membrane treated animals. (C) BALF and serum cytokine expression 24 h post-membrane challenged in male and female mice. Expression of TNF, IL-6, IL-1β, IFNγ, IL-10, and CCL2 by Luminex cytokine panel in male and female BALB/c mice. N = 10 5 M + 5F mice per group. Data represents mean ± SEM. *p < 0.05, **p < 0.01, ***p < 0.001, ****p < 0.0001. Normal data analyzed by 1-Way-ANOVA with Sidak’s multiple comparisons test. Non-normal data (IL-6, IFNγ and IL-10 BALF) analyzed by Kruskal–Wallis test.

Our previous in vitro assays suggested that various SARS-CoV-2 individual proteins elicited varying immune responses. We therefore sought to evaluate the in vivo effects of intranasally (i.n.) administered SARS-CoV-2 membrane protein on both serum and BALF cytokines as well as histological changes in the pulmonary system (Fig. 4A). Here, male and female BALB/c mice were treated i.n. with 15 μg of membrane protein in sterile PBS. Mice showed no significant change in weight or signs of morbidity in the 24 h following i.n. administration (Supplemental Fig. 3A). Compared to vehicle control (PBS) treated mice, membrane treated mice showed increases in the number of nucleated cells isolated from lung lavages in males (p = 0.024) and females (p = 0.15) (Fig. 4B). Cell specific increases of both neutrophils and macrophages were also observed (Supplemental Fig. 3B). Furthermore, in male mice, membrane intranasal treatment significantly increased serum expression of TNF (p = 0.0017), IL-6 (p=0.024), IFNγ (p = 0.0007), IL-10 (p = 0.0001) and CCL2 (p = 0.0003) compared to vehicle control treated animals (Fig. 4C). In female mice, membrane intranasal treatment significantly increased IL-10 expression (p = 0.015). Comparison of male and female mice stimulated with membrane protein showed greater concentrations of TNF (p = 0.0019), CCL2 (p = 0.002) and IL-10 (p = 0.0052) in male mice compared to females. In BALF collected 24 h post-challenge, female mice showed a significant increase in IFNγ (p = 0.017) (Fig. 4C). Significant differences in IFNγ expression levels between male and female mice stimulated with membrane protein were seen, with females showing greater levels of IFNγ than males (p = 0.013).

**Figure 5:**
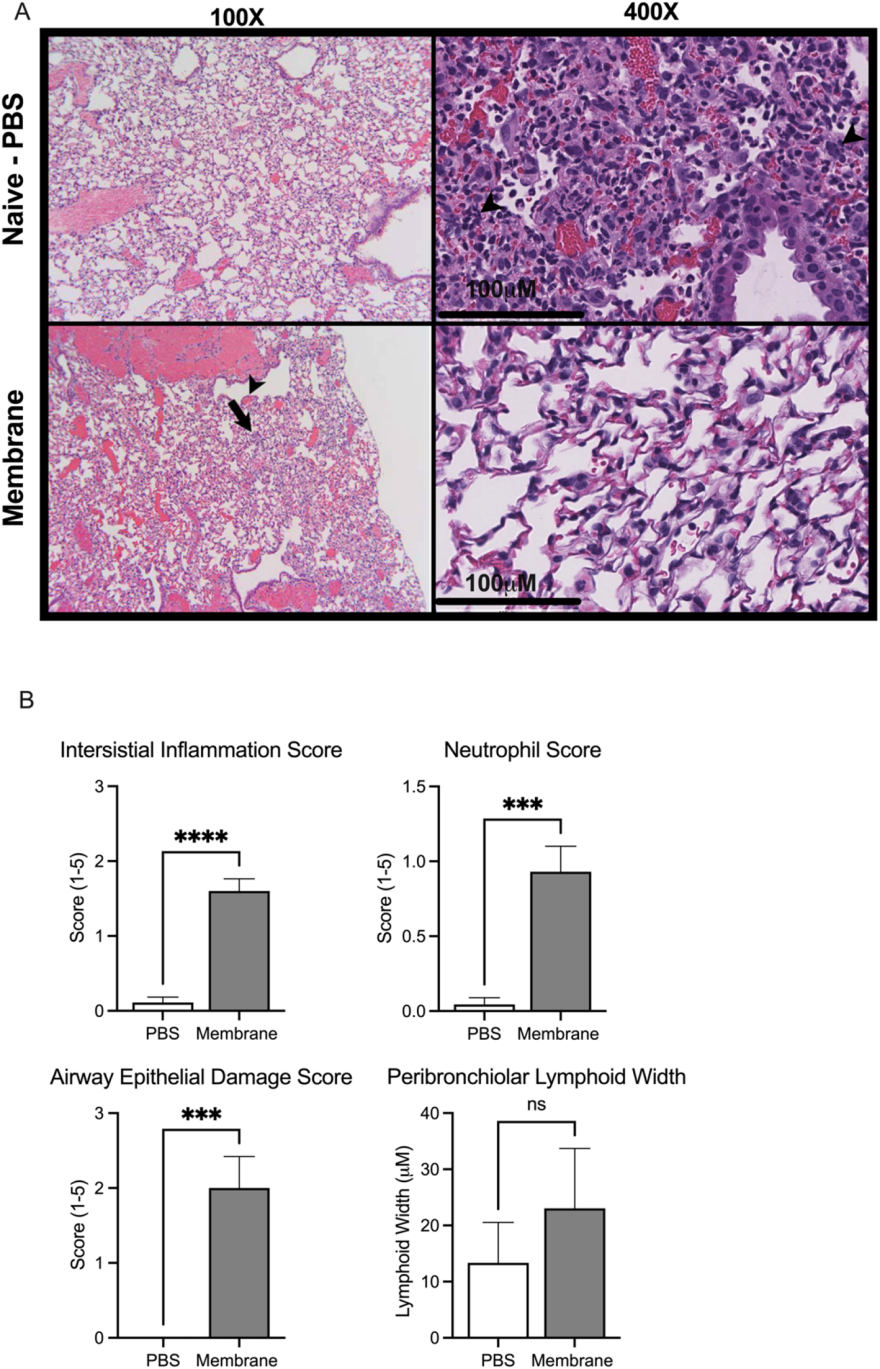
Pulmonary histology of PBS and membrane-treated BALB/c mice. (**A**) Representative photomicrographs (with the approximate mean summed histology score for the group) of lung sections isolated from vehicle control (PBS, i.n.) animals and membrane i.n. treated animals. Arrowheads depict airways epithelial damage and arrow represents interstitial inflammation. (**B**) Membrane i.n. treatment significantly increased interstitial inflammation score, neutrophil score and airway epithelial damage score with modest increases in peribronchiolar lymphoid width observed. Scoring criteria 0–5 indicated in methods section. N = 10 5M + 5F mice per group. ***p < 0.001, ****p < 0.0001. Data analyzed by t-test.

We next evaluated whether the histopathological changes in pulmonary tissue was consistent with the serum and BALF cytokine expression data. Histologic sections from naïve animals were essentially normal, whereas membrane treated animals showed signs of pulmonary inflammation including focal thickening of the alveolar septa by lymphomononuclear inflammatory cells, conspicuous perivascular mixed inflammatory infiltrate, reactive endothelium and fibrin microthrombi (not shown) (Fig. 5A). Quantification of the histological changes showed membrane protein challenge significantly increased the interstitial inflammation score (p < 0.0001), neutrophil score (p < 0.0002), and airway epithelial damage score (p < 0.0003) with a trending increase in peribronchiolar lymphoid width (Fig. 5B). No significant difference was observed between males and females in histology.

## Discussion

The current COVID-19 pandemic has highlighted the lack of appropriate pre-clinical animal models to study SARS-CoV-2 infection. At present, current models fail to accurately recapitulate pulmonary CRS observed in severe COVID-19 patients. The two most common SARS-CoV-2 models using live SARS-CoV-2 are the K18-hACE2 Jackson Laboratories mouse model and Syrian hamster model^9^. In the K18 mouse model, the human ACE-2 gene has been inserted into the genome up to 18 times^10^. Insertion of the hACE-2 gene allows viral entry and subsequent replication in the previously non-infectable mouse model. Although this model allows for active viral replication and has a high death rate within the first week, it often fails to recapitulate CRS observed in long-term and severe COVID-19 infected patients. Furthermore, hACE-2 expression and viral replication in non-pulmonary tissues cause systemic inflammation that is not clinically relevant to patients. In contrast, the Syrian hamster model allows for viral replication and disease that most often does not lead to death and is considered by some to be a milder model of disease^11^. However, similarly this model fails to produce robust viral-induced pulmonary cytokine storm. In addition to the above-mentioned limitations of these models, another major limitation is the need for BSL-3 animal facilities to perform these experiments. It has become evident that the United States is currently underequipped with BSL-3 facilities that can accommodate the needs of all current COVID-19 and as well as other BSL-3 research. This strain has caused facility fees/expenses to skyrocket, limiting our research into the basic mechanisms of SARS-CoV-2 infection, and delaying the potential of new therapeutics from entering the market. Although BSL-3 experiments are still critical to the progress of novel therapeutics, development of novel non-BSL-3 animal models which better recapitulate disease states may alleviate some of the bottle neck issues observed in BSL-3 facilities and provide novel models for the study the longer term pathology SARS-CoV-2 infection.

Here we investigated the immunogenicity of several SARS-CoV-2 proteins as well as characterized the inflammatory response in vivo to these proteins. Our results support that individual SARS-CoV-2 viral proteins illicit varying degrees of immunogenicity evidenced by TNF, IL-6 and IL-1β cytokine production. The structural membrane protein was shown to induce a robust cytokine response both in vitro and in vivo. In vitro, membrane protein challenge with concentrations as low as 1 ng/ml induced TNF expression indicating the potency of this protein in activating immune cells. When challenged intranasally in vivo, membrane protein showed a robust induction of infiltrating immune cells into the pulmonary system accompanied by upregulation of several key cytokines in the blood and BALF. Interestingly, we did observe several different serum cytokine expressions between males and females. Male mice expressed higher levels of inflammatory cytokines TNF, IL-6, CCL2, and anti-inflammatory cytokines IL-10. These sex differences in cytokine expression are of particular interest due to early data in the COVID-19 pandemic showing differential survival rates between male and female patients, in which male patients died at greater rates than females when infected^12,13^. Overtime and SARS-CoV-2 variants, this sex difference has remained consistent in multiple studies reported to date, at least from Western nations. There are exceptions, with India, reporting greater mortality rates in females rather than males^14,15^. Our sex differences observed in response to membrane i.n. challenge support that differential immune response via cytokine signaling may contribute to the severity and disease progression of COVID-19 infected patients.

Clinical data from COVID-19 patients has identified hyperinflammation as a major contributor to disease progression and poor outcomes^16,17^. Initial viral recognition is initiated by the innate immune system, and subsequent refinement of the adaptive immune system leads to the production of antibodies against the virus^18–20^. Critical to this response is the production of inflammatory cytokines which choreograph overall immune cell response. Although inflammation and immune response are critical to viral detection, growing evidence from COVID-19 patients indicates the virus triggers hyperinflammation (i.e., inflammation greater than physiologically needed) which underlies respiratory failure^21^. In particular several cytokines such as TNF and IL-6 have been shown to be upregulated^22,23^. Considered a master regulator of the proinflammatory response, TNF, plays a critical role in activating the innate immune system leading to immune cell infiltration at the site of infection or injury. Our model of SARS-CoV-2 membrane induced pulmonary inflammation shows robust upregulation of TNF mirroring changes observed in clinical patients. Thus, this model has the potential to screen early therapeutics to better understand drug pharmacokinetics as well as measure serum and BALF cytokines changes in response to treatments.

In addition to acting as a novel non-BSL-3 model of COVID-19 viral-induced CRS, our data supports that the individual SARS-CoV-2 proteins may be contributing to the inflammation observed in the lungs of patients. Interestingly, previous work has shown that live SARS-CoV-2 virus administration into non hACE2 expressing rodent strains does not allow for active viral replication and shows minimal inflammation^24^. Although these viruses contain membrane protein within in their virion, multiple factors may impact the lack of immunogenicity often observed in these studies. In these scenarios the membrane protein may be oriented within the virion in which the antigenic domain of the protein is inaccessible for immune detection. Additionally, macrophage phagocytosis of whole virion may prevent individual viral proteins from being free abound within the host tissue and limit their immunogenicity.

However, in hACE2 expressing species as the virus is actively replicating, premature cell lysis or tissue damage can lead to the release of unpackaged viral proteins. These viral proteins have the potential to stimulate a local immune response through their immunogenic properties described in this work. Although the stability of the individual viral proteins in patients is not predicted to be very long, their immediate effect on local tissues can induce a feed forward mechanism in which initial cytokine signaling triggered by their immunogenicity can signal for additional immune cells to infiltrate and provide a longer immune response than the initial inflammatory agonism effect of the proteins. In fact, the expression and immunogenicity of membrane protein is further highlighted by work which shows that anti-membrane antibodies are present in patients up to one year post COVID-19 infection^25^. In addition to the immune response to SARS-CoV-2 membrane protein, previous literature has shown that other SARS-CoV-2 proteins such as envelope protein can also elicit an immune response and changes in tissue pathology. In a study by Xia et al., treatment of intravenous SARS-CoV-2 envelop protein induced upregulation of several inflammatory cytokines in mice^26^. Thus, the contribution of these proteins and future understanding of their distinct downstream signaling pathways in immune cells may open up novel therapeutic approaches targeting COVID-19 viral pulmonary inflammation.

Overall, our results support that individual SARS-CoV-2 proteins are able to illicit a robust immune response in human immune cells. These immunological responses to the various proteins may elucidate novel inflammatory signaling pathways that can be therapeutically targeting in COVID-19. Furthermore, our in vivo work has characterized a novel model of SARS-CoV-2 membrane-induced pulmonary inflammation which can be an alternative approach to research requiring BSL-3 facilities to understand SARS-CoV-2 inflammation.

### Supplementary Information

Supplementary Figures.

## Data Availability

The data sets generated throughout these studies are available from the corresponding author on reasonable request.
