# Supplementary material for: Investigation of SARS-CoV-2 individual proteins reveals the in vitro and in vivo immunogenicity of membrane protein
Source: Sci Rep. 2023 Dec 18;13:22873. doi: 10.1038/s41598-023-49077-2 (PMC10739983; doi:10.1038/s41598-023-49077-2)
Supplement: Supplementary file 1 — Supplementary Figures. [file 41598_2023_49077_MOESM1_ESM.docx]

**In vitro and in vivo immunogenicity of SARS-CoV-2 proteins: highlight on membrane protein**

Timothy Haystead^1,2^, Eric Lee^3^, Kirstin Cho^3^, Gail Gullickson^3^, Philip Hughes^1,2^, Greta Krafsur^3^, Robert Freeze^1^, Scott Scarneo^1*^

**Affiliations:**

^1^ EydisBio Inc. Durham, NC 27701

^2^ Duke University School of Medicine, Department of Pharmacology and Cancer Biology, Durham, NC 27701

^3^ Inotiv Inc, Bolder, CO

*Indicates Corresponding author.

**Corresponding Author:**

Scott Scarneo, PhD

[scott.scarneo@eydisbio.com](mailto:scott.scarneo@eydisbio.com)

**Running title:** Immunogenicity of SARS-CoV-2 membrane protein

**Supplemental Figure 1.** (A) Comparison of 2 vendor SARS-CoV-2 membrane protein immunogenicity, evidenced by TNF levels. Membrane protein purchased from both Novus and Tocris were treated at 50ng/mL on PMA differentiated THP-1 cells for 24 hours. TNF expression levels determined in supernatant samples. Untreated THP-1 cells were included as a control group (Naïve). (B) Cell survival 24 hours post membrane treatment compared to non-treated control cells. N=8/group. Data represents mean±SEM.

**Supplemental Figure 2.** IFNγ and IL-4 expression levels in differentiated THP-1 cells following SARS-CoV-2 membrane treatment.

**Supplemental Figure 3.** (A) Male and female mice weights prior to (baseline) and 24 hours post 15μg membrane challenge. N=5/group/sex. (B) Neutrophil and macrophages infiltrates in BALF from mice 24 hours post PBS or membrane challenge. Data represents mean±SEM. Data analyzed by 2-Way-ANOVA with Sidaks multiple comparisons.
